# Extra-Wide Lane Ambiguity Resolution and Validation for a Single Epoch Based on the Triple-Frequency BeiDou Navigation Satellite System

**DOI:** 10.3390/s20051534

**Published:** 2020-03-10

**Authors:** Jian Deng, Aiguo Zhang, Nenghui Zhu, Fuyang Ke

**Affiliations:** 1 Department of Surveying and Remote Sensing Engineering, Xiamen University of Technology, Xiamen 361024, China; dengjian@xmut.edu.cn (J.D.); zhangaiguo@xmut.edu.cn (A.Z.); 2 Big Data Institute of Digital Natural Disaster Monitoring in Fujian, Xiamen University of Technology, Xiamen 361024, China; 3 School of Applied Mathematics, Xiamen University of Technology, Xiamen 361024, China; zhunenghui@xmut.edu.cn; 4 School of Remote Sensing and Geomatics Engineering, Nanjing University of Information Science and Technology, Nanjing 210044, China

**Keywords:** EWL ambiguity resolution, reliability, triple-frequency, gross error detect, BDS

## Abstract

The ambiguity resolution (AR) and validation of the global navigation satellite system (GNSS) have been challenging tasks for some decades. Considering the reliability problem of extra-wide-lane (EWL) ambiguity in the triple-carrier ambiguity resolution (TCAR), a method for validating the reliability of the EWL ambiguity using a single epoch was proposed for the BeiDou Navigation Satellite System (BDS). For the initial EWL ambiguity, obtained using a rounding estimator with a geometry-free (GF) model, the double-difference ionospheric delay was first estimated to construct a relative positioning model with an initial fixed ambiguity. Second, based on the theory of gross error detection and the AR characteristics of EWL, the second-best ambiguity candidate was constructed. Finally, among the two sets of ambiguities, the one with the smaller posterior variance was taken as the reliable ambiguity. The study showed that, for a single epoch, when only one or two satellites had incorrect ambiguities, the AR success rate after ambiguity validation and correction could reach 100% for medium baselines. For long baselines, due to the increase of atmospheric error, the validation was affected to some extent. However, the AR success rates for two long baselines increased from 96.82% and 98.44% to 98.80% and 99.67%, respectively.

## 1. Introduction

Resolving carrier phase ambiguities quickly and reliably is very important for ensuring the positioning timeliness and accuracy, or for developing new fields in high-precision dynamic positioning applications with the global navigation satellite system (GNSS). For a long time, many ambiguity resolution (AR) methods were based on the least-squares (LS) estimation [1,2,3,4], where the float solution and variance-covariance can be obtained and further adjusted to an integer value through some certain estimator, such as rounding [5], bootstrapping [6], and integer LS [7]. These methods have improved the reliability of ambiguity resolution to a certain extent and greatly promoted the development of GNSS real-time and high-precision positioning.

In recent years, with the modernization of the GNSS, more multi-frequency signals will become available for end users. With multi-frequency signals, more useful combinations can be formed, which will benefit AR. The most representative methods are triple-carrier ambiguity resolution (TCAR) [8,9] and cascade integer resolution (CIR) [10,11]. The basic principle of both approaches is essentially the same. The approach starts with the easy-to-fix, extra-wide lane (EWL) combination and steps to the shorter wavelength wide-lane (WL) and narrow-lane (NL) combinations sequentially, whereby the WL combination is used to bridge the longest wavelength EWL and the shortest wavelength NL. Following on from these studies, a large amount of work has been carried out on triple-frequency ambiguity resolution using the TCAR/CIR or modified TCAR/CIR methods [12,13,14,15].

Given that the ambiguity is fixed step by step, the accuracy and reliability of EWL AR is the premise and foundation, which will directly affect the WL/NL AR and the positioning result. For EWL AR, generally, the geometry-free (GF) and the geometry-based (GB) models may be used. The general GF model can be formed as the linear combination between virtual code and phase measurements, or between two phase measurements to eliminate or reduce the geometry-related terms. Feng et al. [16] described a general model using triple-frequency simultaneous measurements to obtain better GF combinations. To further reduce the influence of ionospheric delay, many studies have constructed a geometry- and ionosphere-free (GIF) model to obtain a better AR success rate [17,18,19]. For the general GB model, which can be formed with phase measurements, it is important to note that this model includes the effects of orbital, ionospheric, and tropospheric biases, as well as phase noises. However, the model is usually used in network real-time kinematic (RTK) positioning as the inter-station baseline parameters are precisely known and can be exploited to benefit the estimation of other parameters. Feng identified the three most useful combinations for each of the three frequency GNSS services based on the total noise level [20]. Furthermore, many studies [21,22,23] have discussed the optimal combinations that are suitable for the GB model under different error budget assumptions. Similar to the GIF model, to eliminate the double-difference (DD) ionospheric delays, Gao et al. [23] derived a modified ionosphere-free model for the second EWL/WL AR. In general, the above two types of models usually achieve a higher success rate due to the long wavelengths of the EWL.

The previous studies have demonstrated that the signals of the additional frequency can improve the AR performance, but the AR success rate for the majority of combined EWL observations has not attained 100%, especially in the case of long baselines [19,23,24]. However, given that the ambiguity is an unknown parameter, there is no truth value for the reference in the actual application to validate its accuracy. An incorrect integer ambiguity solution, if overlooked, may cause severe bias in the fixed solution. Thus, the development of a reliable procedure for ambiguity validation is essential. This has proved to be a challenging task for some decades now and is still far from being resolved [25,26]. For the ambiguity validation, the most widely used method is to compare the minimum quadratic form of the residuals and the second quadratic form of the residuals in different ways. The commonly used methods are the F-ratio test [27], the R-ratio test [28], the W-ratio test [29], the difference test [30], and the projector test [31]. The question is how to choose the critical value? Different values have been proposed based on empirical results; however, such values seem only able, for the most part, to give good performance for the given data and the specific measurements considered. To choose proper critical values for the ratio test, Hou et al. [32] proposed the fixed failure-rate ratio test (FFRT), which generated critical values according to user-defined tolerable failure rates. Teunissen and Verhagen pointed out that the critical value should be different with the different measurement models and observation conditions; he then studied the R-ratio test based on the fixed failure rate and developed the so-called “look-up table” for ambiguity validation [33,34,35]. However, this table is not universal and is very computationally time-consuming [26]. Instead of using an empirical constant detection threshold or a fixed failure/success rate requirement in the ratio tests for ambiguity validation, Li et al. [36] proposed an integrity monitoring-based ratio test, which used the ambiguity protection level to control the false alarm and missed detection errors.

The above methods of ambiguity validation are based mainly on the float ambiguity and variance obtained through the LS method. However, the integer rounding estimator is always used for the EWL ambiguity resolution as the effect of noise concerning the wavelength of the combined observation is sufficiently small; that is, we cannot calculate the variance of the ambiguity float, which is different from the LS estimation. Therefore, a routine statistical test method for validating the ambiguity is generally not applicable. Some studies have analyzed the AR success rate of EWL based on prior information of the observation noise and atmospheric error and some useful conclusions have been reached [18,19,23]. However, a priori knowledge of observation bias is indispensable for the evaluation of the success rate of TCAR, but this is difficult to acquire in real-world applications. The reliability of the success-rate-based evaluation method is therefore limited by the unknown bias [37].

To obtain a reliable ambiguity validation of TCAR, based on the theory of gross error detection, this study aimed to study an EWL ambiguity validation method in real-time. The paper is organized as follows: Section 2 presents the basic equations and definitions of triple-frequency BDS observation, and in Section 3, the method for EWL AR for observation $\Delta \phi_{\left(1,4,-5\right)}$ is analyzed. Section 4 gives some discussion on the EWL ambiguity validation. Section 5 presents a method for EWL ambiguity validation based on gross error detection. In Section 6, the results of several sets of experiments are presented. Finally, the conclusions are summarized.

## 2. Basic Equations and Definitions of Triple-Frequency BDS Observations 

For triple-frequency signals, the combined double-difference (DD) observations of the code P and the carrier phase ϕ in meters (m) can be expressed separately as follows:

(1) $$\Delta P_{\left(i,j,k\right)}=\Delta \rho +\Delta T+\beta_{\left(i,j,k\right)}\Delta I+\varepsilon_{\Delta P(i,j,k)},$$

(2) $$\Delta \phi_{\left(i,j,k\right)}=\Delta \rho +\Delta T-\beta_{\left(i,j,k\right)}\Delta I+\lambda_{\left(i,j,k\right)}\Delta N_{\left(i,j,k\right)}+\varepsilon_{\Delta \phi (i,j,k)},$$

where Δ is the DD operator and the subscripts (*i,j,k*) represent the frequencies used in the combination [21]. ρ denotes the distance between the satellite and the receiver, and *T* and *I* are the tropospheric delay and ionospheric delay, respectively. The combined DD code and phase can be expressed as:

(3) $$\Delta P_{\left(i,j,k\right)}=\frac{i\cdot f_{1}\cdot \Delta P_{1}+j\cdot f_{2}\cdot \Delta P_{2}+k\cdot f_{3}\cdot \Delta P_{3}}{i\cdot f_{1}+j\cdot f_{2}+k\cdot f_{3}},$$

(4) $$\Delta \phi_{\left(i,j,k\right)}=\frac{i\cdot f_{1}\cdot \Delta \phi_{1}+j\cdot f_{2}\cdot \Delta \phi_{2}+k\cdot f_{3}\cdot \Delta \phi_{3}}{i\cdot f_{1}+j\cdot f_{2}+k\cdot f_{3}},$$

where the symbols $\Delta P_{i}$ and $\Delta \phi_{i}$ are the DD code and phase measurements of the distance for the *i*th frequency $f_{i}$ [38]. λ and N represent the combined wavelength and integer ambiguity of the carrier phase, which can be expressed as:

(5) $$\left\{ \begin{array}{l} \lambda_{\left(i,j,k\right)}=\frac{C}{i\cdot f_{1}+j\cdot f_{2}+k\cdot f_{3}}\\ \Delta N_{\left(i,j,k\right)}=i\Delta N_{1}+j\Delta N_{2}+k\Delta N_{3} \end{array}\right.,$$

where *C* is the speed of light and the ionospheric scale factors (ISF) β(i,j,k) are defined as:

(6) $$\beta (i,j,k)=\frac{f_{1}^{2}(i/f_{1}+j/f_{2}+k/f_{3})}{i\cdot f_{1}+j\cdot f_{2}+k\cdot f_{3}},$$

and where $\varepsilon_{\Delta P(i,j,k)}$ and $\varepsilon_{\Delta \phi (i,j,k)}$ refer to the noise of the combined code and phase observation, respectively, in meters. Assuming the three carrier measurements have the same precisions, that is, $\sigma_{\Delta \phi_{1}}=\sigma_{\Delta \phi_{2}}=\sigma_{\Delta \phi_{3}}=\sigma_{\Delta \phi }$, then the standard deviation (STD) of the combined phase noise $\varepsilon_{\Delta \phi (i,j,k)}$ can be expressed as follows:

(7) $$\sigma_{\Delta \phi (i,j,k)}=\frac{\sqrt{{\left(i\cdot f_{1}\right)}^{2}+{\left(j\cdot f_{2}\right)}^{2}+{\left(k\cdot f_{3}\right)}^{2}}}{i\cdot f_{1}+j\cdot f_{2}+k\cdot f_{3}}\sigma_{\Delta \phi }=\mu_{\Delta \phi (i,j,k)}\cdot \sigma_{\Delta \phi },$$

where μ denotes the noise scale factors (NSFs). For the pseudo-range of the BeiDou Navigation Satellite System (BDS), the code chipping rate on B3 is different from those on B1 and B2, assuming that $\sigma_{\Delta P_{1}}=\sigma_{\Delta P_{2}}=5\sigma_{\Delta P_{3}}=\sigma_{\Delta P}$ [22], and the STD of the combined phase noise $\varepsilon_{\Delta P(i,j,k)}$ is given as:

(8) $$\sigma_{\Delta P(i,j,k)}=\frac{\sqrt{{\left(i\cdot f_{1}\right)}^{2}+{\left(j\cdot f_{2}\right)}^{2}+{\left(0.2\cdot k\cdot f_{3}\right)}^{2}}}{i\cdot f_{1}+j\cdot f_{2}+k\cdot f_{3}}\sigma_{\Delta P}=\mu_{\Delta P(i,j,k)}\cdot \sigma_{\Delta P}.$$

## 3. AR Model for EWL Observation $\Delta \phi_{\left(1,4,-5\right)}$

From Equations (5) to (8) given above, it can be seen that different combinations of *i*, *j*, and *k* correspond to different errors. As for AR, given the long wavelength of the EWL combination, the ionospheric delay and observation noise are ignored here, and a rounding strategy is employed to fix the ambiguity to produce a high success rate. Moreover, among all the EWL and WL combinations, as long as the sum of the combined coefficients is zero, that is, i+j+k=0, only two are independent [20,21]; in other words, we can use two groups of ambiguities with a high success rate to deduce the ambiguities of other linear-correlation combined observations using a simple transformation. According to Tang et al. [22] and Li et al. [39], two groups of optimal coefficients of EWL combinations for BDS are (0, −1, 1) and (1, 4, −5), whose wavelengths are 4.48 m and 6.37 m, respectively. Thus, a fixed solution for the EWL ambiguity with a high success rate can be obtained via rounding. Many researchers have studied this aspect and proposed several very reliable solutions [15,19,23,24].

For the ambiguity $\Delta N_{\left(0,-1,1\right)}$, the GIF mode can be formed with EWL observation $\Delta \phi_{\left(0,-1,1\right)}$ and $\Delta P_{\left(0,1,1\right)}$, and the ambiguity can be easily fixed using a single epoch, that is:

(9) $$\Delta N_{\left(0,-1,1\right)}=\left[\frac{\Delta \phi_{\left(0,-1,1\right)}-\Delta P_{\left(0,1,1\right)}}{\lambda_{\left(0,-1,1\right)}}\right]$$

where [⋅] represents the rounding operator. The AR accuracy is only affected by the observation noise of the carrier and the pseudo-range. Experiments showed that even when the observation noise is large, for example, $\sigma_{\Delta \phi }=0.01\;m$ and $\sigma_{\Delta P}=1\;m$, the success rate can reach 100%, which is more reliable, and thus it can be used in the first step of all TCAR methods [19,23,24]. Therefore, this study focused on the ambiguity resolution and validation for EWL observation $\Delta \phi_{\left(1,4,-5\right)}$. Generally, GB and GF models are available for EWL AR. The GB model is more commonly used for AR between network RTK reference stations. This model is based on the LS principle and can be combined with least-square ambiguity decorrelation adjustment (LAMBDA) to validate ambiguity reliability [21,22], which is similar to the traditional method for a dual frequency and will not be discussed in detail here. As for the GF model, concerning Equations (1) and (2), the general expression for the ambiguity solution is:

(10) $$\Delta N_{\left(1,4,-5\right)}=\frac{1}{\lambda_{\left(1,4,-5\right)}}[\Delta \phi_{\left(1,4,-5\right)}-\Delta P_{\left(i,j,k\right)}+(\beta_{\left(1,4,-5\right)}+\beta_{\left(i,j,k\right)})\Delta I].$$

The ambiguity is mainly affected by the observed noise and the ionospheric delay, which are usually ignored in the actual solution. Therefore, the STD σ and the systematic bias δ of the float ambiguity estimations can be derived according to the variance-covariance propagation laws, which are expressed as:

(11) $$\sigma_{\Delta N_{\left(1,4,-5\right)}}=\frac{1}{\lambda_{\left(1,4,-5\right)}}\sqrt{\mu_{\Delta \phi (1,4,-5)}^{2}\sigma_{\Delta \phi }^{2}+\mu_{\Delta P(i,j,k)}^{2}\sigma_{\Delta p}^{2}},$$

(12) $$\delta_{\Delta N_{\left(1,4,-5\right)}}=\frac{(\beta_{\left(1,4,-5\right)}+\beta_{\left(i,j,k\right)})\Delta I}{\lambda_{\left(1,4,-5\right)}}.$$

Then, the float ambiguity estimations obey the following distribution:

(13) $$\Delta N_{\left(1,4,-5\right)}∼N(\delta_{\Delta N_{\left(1,4,-5\right)}},\sigma_{\Delta N_{\left(1,4,-5\right)}}).$$

The AR success rate, known as the AR reliability, is defined as the percentage of the correctly solved epoch numbers out of the total epoch number [22]. Here, the AR success rate with a rounding can be theoretically computed using Equation (14):

(14) $$P(-0.5<x<0.5)=\int_{-0.5}^{+0.5}\frac{1}{\sigma \sqrt{2\pi }}\exp(-\frac{{\left(x-\delta \right)}^{2}}{2\sigma^{2}})dx.$$

It can be seen that with different combinations of observations, the STD σ and the systematic bias δ are different, and thus the AR success rates are also different. We can select optimal pseudo-range combination observations with the minimum observed noise to construct the GF-IF model that can meet the following requirements:

(15) $$\left\{ \begin{array}{l} \beta_{\left(1,4,-5\right)}+\beta_{\left(i,j,k\right)}=0\\ \sigma_{\Delta N_{\left(1,4,-5\right)}}=\min \end{array}\right..$$

Assuming the search space of each combination coefficient *i*, *j*, and *k* is limited to [−10, 10], the traditional ergodic optimization method is adopted to obtain the optimal observation $\Delta P_{\left(-5,2,2.65\right)}$ with the minimum NSF $\mu_{(-5,2,2.65)}=-4.039$. In this study, we also selected the common observations $\Delta P_{\left(1,0,0\right)}$ and $\Delta P_{\left(0,1,1\right)}$ for AR; moreover, after the $\Delta \phi_{\left(0,-1,1\right)}$ ambiguities are resolved, the ambiguity-fixed EWL observations can be regarded as precise “pseudo-range” observations to support the resolution of the second EWL/WL ambiguity with GF mode, and the ambiguity $\Delta N_{\left(1,4,-5\right)}$ can be expressed as:

(16) $$\Delta N_{\left(1,4,-5\right)}=\frac{1}{\lambda_{\left(1,4,-5\right)}}[\Delta \phi_{\left(1,4,-5\right)}-\Delta \phi_{\left(0,-1,1\right)}+\lambda_{\left(0,-1,1\right)}\cdot \Delta N_{\left(0,-1,1\right)}+(\beta_{\left(1,4,-5\right)}-\beta_{\left(0,-1,1\right)})\cdot \Delta I].$$

With the above four combined observations, we can obtain the EWL ambiguity $\Delta N_{\left(1,4,-5\right)}$, and the corresponding STDs and ISFs for four different cases are shown in Table 1.

**Figure 1:**
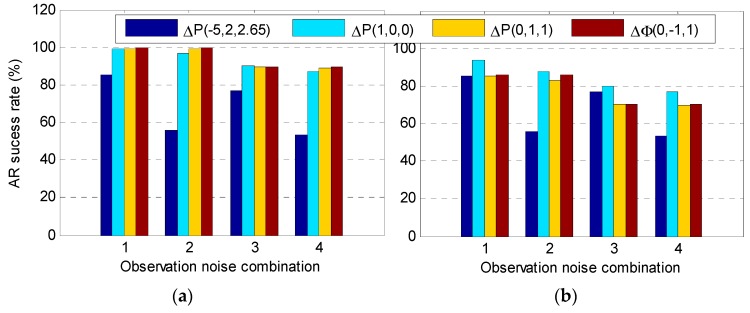
Ambiguity resolution (AR) success rate using four observations with different noise levels and double-difference (DD) ionospheric delays: (**a**) DD ionospheric delay ΔI=40cm and (**b**) DD ionospheric delay ΔI=100cm.

According to Equation (12), the systematic bias σ also depends on the effect of the ionospheric delay. For the 20–100-km medium–long baseline, the first-order ionospheric delay is generally <40 cm, and for the 100–500-km long baseline the ionospheric delay is <100 cm [20,22]; therefore, by obtaining ΔI=40 cm and ΔI=100 cm using Equation (14), the AR success rate with the above four observations at different ionospheric errors and observation noises were calculated, where the results are shown in Figure 1.

It can be seen that when the DD ionospheric delay was 40 cm, for the ambiguity $\Delta N_{\left(1,4,-5\right)}$, the observations $\Delta P_{\left(1,0,0\right)}$, $\Delta P_{\left(0,1,1\right)}$, and $\Delta \phi_{\left(0,-1,1\right)}$ attained a similar success rate for the four kinds of observation noise levels, while the optimal observation $\Delta P_{\left(-5,2,2.65\right)}$ was the lowest; that is, although the GF-IF model was not affected by the ionospheric delay, it also amplified the observation noise, especially when the pseudo-range STD was large (in cases 2 and 4) such that the AR success rate was lower. If the DD ionospheric delay was 100 cm, the ambiguity success rate based on the four combined observations was less than 90% with different observation noises. In general, the ambiguity success rate obtained using observation $\Delta P_{\left(1,0,0\right)}$ was relatively high, which was mainly due to the small ionospheric scale factor (ISF). Therefore, in practical applications, when the ionospheric delay is large, such as in long-baseline or low-latitude regions, this combination can be used to obtain the EWL ambiguity with a higher success rate.

**Figure 2:**
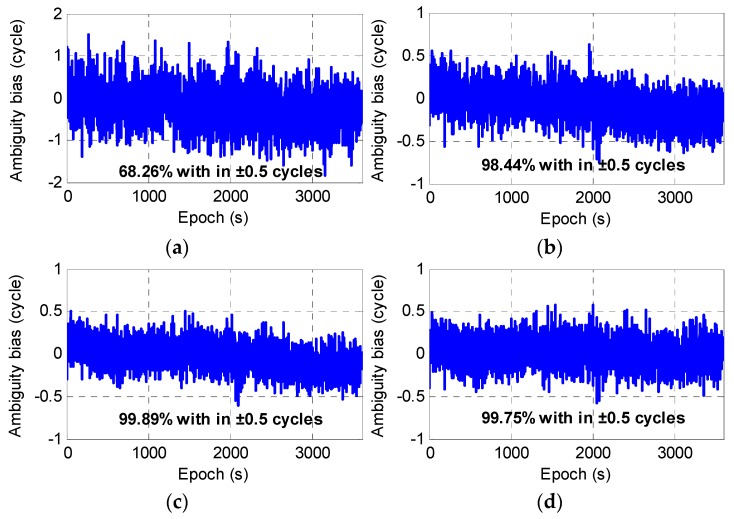
Ambiguity $\Delta N_{\left(1,4,-5\right)}$ biases with four different combined observations: (**a**) using observation $\Delta P_{\left(-5,2,2.65\right)}$, (**b**) using observation $\Delta P_{\left(1,0,0\right)}$, (**c**) using observation $\Delta P_{\left(0,1,1\right)}$, and (**d**) using observation $\Delta \phi_{\left(0,-1,1\right)}$.

The above analysis was mainly based on the empirical STD and the DD ionospheric delay in order to analyze and estimate the success rate of the EWL ambiguity $\Delta N_{\left(1,4,-5\right)}$. To further compare and analyze the actual effect of the AR using the four observations, two reference stations SQXY and XYXX with a separation distance of 175 km were selected from the Henan continuously operating reference station (CORS), China. A total of 3600 epochs of data were collected with a 1 s sampling interval on 1 March 2016; a total of eight BDS satellites with an elevation cut-off angle of 15° were available for use. The above four combined observations were used to solve the ambiguity $\Delta N_{\left(1,4,-5\right)}$ using a single epoch. Due to the long wavelength used for the combined observations, the AR success rates of most satellites were 100%, and satellite C05 had the lowest accuracy. Figure 2 shows the single-epoch biases obtained by comparing the float ambiguities and their true values. In general, similar to the analysis results based on the empirical value estimation, the AR success rate with the optimal observation $\Delta P_{\left(-5,2,2.65\right)}$ was the lowest and only 68.26% were biases within ±0.5 cycles. That is, nearly one-third of the ambiguity integer estimates were incorrect. For the other three observations, the results were about the same, with the success rate exceeding 98%. The observation $\Delta P_{\left(0,1,1\right)}$ gave the best result with a success rate of 99.89%.

## 4. Discussion on the Method of EWL Ambiguity Validation

**Figure 3:**
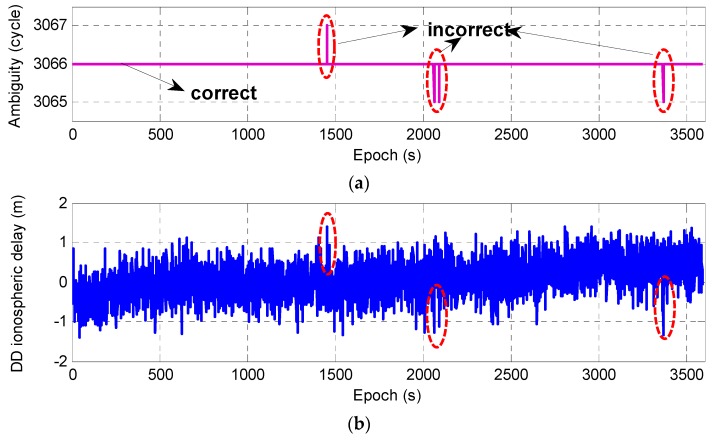
Performance of fixed ambiguity (**a**) and DD ionospheric delay (**b**) for satellite C05.

From the above theoretical and experimental analysis, it can be seen that although the ambiguity $\Delta N_{\left(1,4,-5\right)}$ had a high success rate, due to the influence of observation noise and the ionospheric delay, 100% accuracy could not be guaranteed for AR in a single-epoch. For the TCAR method, the incorrect EWL ambiguity will affect the AR for the NL or the basic frequency observation, and eventually lead to a poor positioning result. Therefore, it is very important to ensure the reliability of the EWL ambiguity in real-time. Here, we will discuss some strategies to ensure the validity of the ambiguity. Taking the AR with observation $\Delta P_{\left(0,1,1\right)}$ as an example, the same experimental data as in the previous section is adopted. Figure 3 (top) shows the single-epoch ambiguity performance of satellite C05, where four epochs have incorrect ambiguities. In practical application, for the post-processing solution, the average value can be taken as the final accurate value to ensure the reliability of the ambiguity [21]. For a real-time solution, the accuracy of the subsequent ambiguity can be checked by averaging the epochs one by one or comparing integer values between two consecutive epochs [40], but this relies mainly on prior observation information. It is, however, necessary to ensure the continuity of the carrier observation and to avoid cycle slipping; moreover, this method cannot effectively be used in the initial epoch. Considering that the DD ionospheric delay can be reversed through the fixed ambiguity, we can further validate the reliability of ambiguity using the estimated ionospheric delay. Based on the fixed EWL ambiguity, the GF model can be formed with observations $\Delta P_{\left(i,j,k\right)}$ and $\Delta \phi_{\left(1,4,-5\right)}$, and the DD ionospheric delay may be expressed as follows:

(17) $$\Delta I=\frac{1}{\beta_{\Delta P(i,j,k)}+\beta_{\Delta \phi (1,4,-5)}}(\Delta P_{\left(i,j,k\right)}-\Delta \phi_{\left(1,4,-5\right)}+\lambda_{\left(1,4,-5\right)}\Delta N_{\left(1,4,-5\right)}).$$

From Equation (17), assuming the combined pseudo-range $\Delta P_{\left(0,1,1\right)}$ was selected, when there was one cycle error in the ambiguity, the DD ionospheric delay caused a bias of nearly 3 m. Figure 3b shows the DD ionospheric delay for each epoch for satellite C05. It can be seen that when the ambiguity was incorrect, there was some abnormality in the corresponding DD ionospheric delay. However, due to the large noise and randomness of the observation, the abnormal values were not very prominent in the experiment. Therefore, it was difficult to give an appropriate threshold to validate the reliability of the ambiguity through ionospheric outliers.

In this paper, based on the theory of gross error detection, according to the AR characteristics of the EWL, we took the influence of the incorrect ambiguity on the observation as a gross error, with the principle of the smallest posterior variance being used to realize the validation of the EWL ambiguity.

## 5. Ambiguity Validation Based on Gross Error Detection

With an estimated DD ionospheric delay and fixed EWL ambiguity, a model for relative positioning was constructed. For relative positioning, the coordinates of the base station A are usually known. As a point to be determined, the initial coordinates of station B can generally be obtained using single-point positioning, assuming the initial coordinates are ${(x}_{0}{,y}_{0}{,z}_{0})$ and the corresponding correction is (δx,δy,δz). For the tropospheric delay, this can be expressed as the product of the zenith total delay (ZTD) and the mapping function (MF), which is a function of the elevation angle of the satellite. The ZTD is composed of the zenith hydrostatic delay (Zhd) and the zenith wet delay (Zwd). The dry component Zhd is estimated through the global pressure and temperature (GPT) model, while the wet component Zwd is estimated as unknown parameters in the observation model. As for the mapping function, the Hopfield model was adopted here. The error equation is obtained after linearization, that is:

(18) V=B•X−L,

where $B=[\begin{array}{lllll} \nabla l & \nabla m & \nabla n & \nabla MF(E_{A}) & -\nabla MF(E_{B}) \end{array}]$; ∇ is the single difference (SD) operator between satellites; l,m,n are the linearization coefficients in all directions; *E* represents the elevation of the satellite; and $X=[\begin{array}{ccccc} \delta x & \delta y & \delta z & Zwd_{A} & Zwd_{B} \end{array}{]}^{\prime }$. L can be expressed as:

(19) $$L=-(\Delta \rho^{0}-\Delta \phi_{\left(1,4,-5\right)}+\lambda_{\left(1,4,-5\right)}\Delta N_{\left(1,4,-5\right)}-\beta_{\left(1,4,-5\right)}\Delta I+\Delta T_{dry})$$

where $\rho_{0}$ denotes the distance between the satellite and the receiver with the initial coordinate value of station *B*, and $T_{dry}$ is the dry component of the tropospheric delay. 

Assuming that the weighting of each satellite observation is the same, the parameter estimates and the residuals can be obtained through the LS method. The residual can reflect the quality of the corresponding observations to a certain extent, and we considered the influence of the incorrect ambiguity on the observation as a gross error. Combining Equations (17) and (19), it was deduced that when the ambiguity $\Delta N_{\left(1,4,-5\right)}$ has a δN-cycles error, the added gross error δL in the observation may be expressed as:

(20) $$\delta L=-\beta_{\left(1,4,-5\right)}\cdot \frac{\lambda_{\left(1,4,-5\right)}\cdot \delta N}{\beta_{\Delta P(i,j,k)}+\beta_{\left(1,4,-5\right)}}+\lambda_{\left(1,4,-5\right)}\cdot \delta N,$$

where the first term on the right side of the equation is the influence of the incorrect ambiguity on the DD ionospheric delay. For example, if the observation $\Delta P_{\left(0,1,1\right)}$ is used to estimate the DD ionospheric delay, when δN=±1 cycle, the gross error δL will be ±4.519 m, which will affect the residuals of all observations to different degrees; that is, we can validate the ambiguity using the residual. The standardized residual is more commonly used and can be denoted as:

(21) $$e_{i}=\frac{\left|v_{i}\right|}{\sigma_{0}\sqrt{r_{i}}},$$

where $\sigma_{0}^{2}$ is the a priori variance and $r_{i}$ is the redundancy number. If the observations have equal weight, the matrix *R* of the redundancy number can be expressed as:

(22) $$R=I-B\cdot {\left(B^{T}\cdot B\right)}^{-1}\cdot B^{T},$$

where *I* is the identity matrix. When the number of observations with gross errors is *q*, the effect on the *i*th residual ${\hat{v}}_{i}$ is:

(23) $${\hat{v}}_{i}=-r_{i1}\Delta_{1}-r_{i2}\Delta_{2}-\ldots -r_{iq}\Delta_{q}.$$

That is, the residual ${\hat{v}}_{i}$ contains the influence of all the gross errors, the size of which depends on the redundancy number corresponding to each gross error. In particular, if only one observation has a gross error, the impact of this gross error on each residual is ${\hat{v}}_{i}=-r_{ij}\Delta_{j}$. The experiment showed that $r_{ii}>r_{ij}$; that is, the gross error affected its own residuals much more than other residuals. Similarly, the corresponding standardized residuals may have a greater probability of being larger than others. However, when multiple observations contain gross errors, the effects of the gross errors on the residuals may cancel each other out. At this time, regarding the residuals, it is more difficult to reflect on whether the observations contain gross errors, but as long as only one or two observations contain a gross error, it is feasible to detect the gross error from the abnormal residuals [41]. At this point, it is generally the case that the first two gross errors that are detected are responsible. Moreover, the EWL ambiguity resolutions for each satellite are independent of each other, and they also have a high success rate, as was the case in the previous section. Furthermore, the probability that many ambiguities are incorrect in the same epoch is generally small. Therefore, in a single epoch, when only one or two satellites have incorrect ambiguities, it is feasible to perform ambiguity validation using gross error detection.

**Figure 4:**
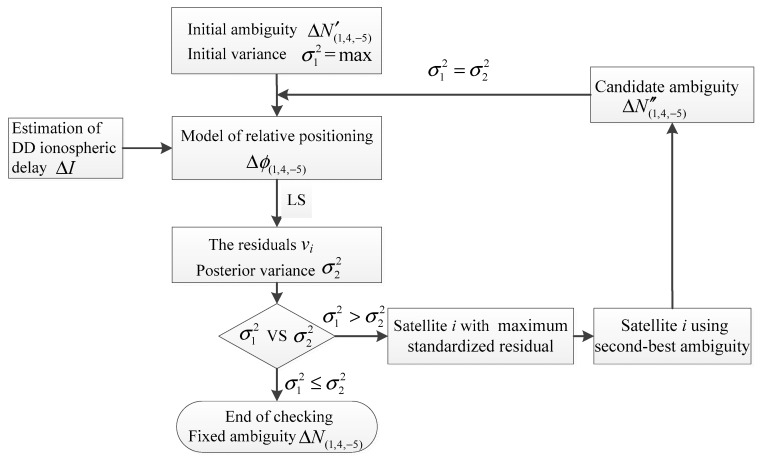
Method for validating the reliability of the EWL ambiguity $\Delta N_{\left(1,4,-5\right)}$. LS: Least-squares estimation.

However, for gross error detection, it is difficult to specify a suitable threshold for standardized residuals to validate whether the observation contains a gross error. Due to the long wavelength of EWL observation, the total error of AR is generally within 0.5 cycles, and the fixed ambiguity with the highest reliability is usually the two that are nearest to the floating ambiguity. For example, if the floating ambiguity is 8.3 cycles, the maximum probability of the fixed value is 8 or 9, while it is almost impossible to be 7 or 10 cycles because this would mean that the total error of the observation reaches 1.3 cycles (8.2 m) or 1.7 cycles (10.8 m). For this reason, we can choose the two ambiguities with the highest reliability as the candidate values and further determine the exact one by comparing the posterior variance. Based on the above analysis, this study proposed a method for validating the reliability of the EWL ambiguity $\Delta N_{\left(1,4,-5\right)}$, as shown in Figure 4. The approach mainly relied on the following steps:

Step 1: The initial fixed ambiguity $\Delta {N^{\prime }}_{\left(1,4,-5\right)}$ is obtained based on the GF model.

Step 2: The DD ionospheric delay is estimated, and the model of the relative positioning is constructed with the estimated DD ionospheric delay and the initial fixed ambiguity.

Step 3: The residual $v_{i}$ and the posterior variance $\sigma_{1}^{2}$ are obtained through the LS method. Assuming that the initial ambiguity corresponding to the maximum standardized residual is incorrect and replaced by the second-best ambiguity of this observation, while the other ambiguity values are unchanged, a new set of candidate ambiguities $\Delta {N^{\prime \prime }}_{\left(1,4,-5\right)}$ can be reconstructed. The new posterior variance $\sigma_{2}^{2}$ will be obtained through the LS method.

Step 4: When comparing the two posterior variance values, if $\sigma_{1}^{2}\leq \sigma_{2}^{2}$, it is considered that the original hypothesis is wrong and there is no gross error in the observation; that is, the initial ambiguity $\Delta {N^{\prime }}_{\left(1,4,-5\right)}$ is correct. On the contrary, if the original hypothesis is established, the candidate ambiguity $\Delta {N^{\prime \prime }}_{\left(1,4,-5\right)}$ is selected as the accurate value and the process returns to step 2 to continue to validate the reliability of other ambiguities.

## 6. Experiments and Analysis

### 6.1. Medium–Long Baseline

**Figure 5:**
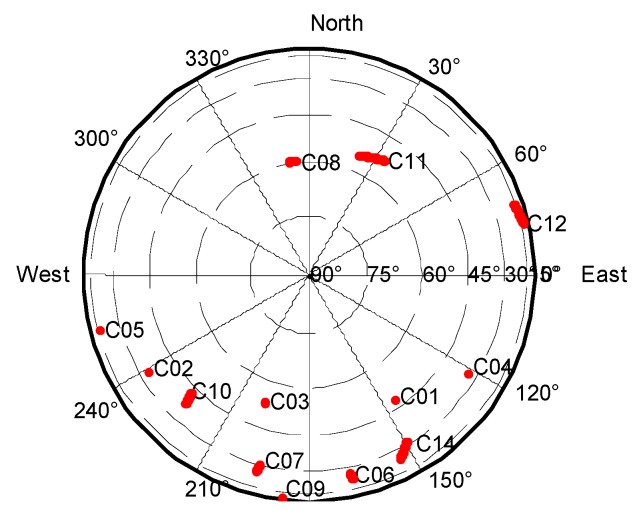
Distribution of BeiDou Navigation Satellite System (BDS) satellites at station TAOY.

Two reference stations, TAOY and JINT, with a separation distance of 66 km were selected from the Suzhou CORS, China. A total of 1800 epochs of data were collected with a 1 s sampling interval on 18 December 2014. Figure 5 shows the distribution of BDS satellites visible from reference station TAOY during this period. It can be seen that a total of 12 BDS satellites were observed with a cut-off elevation angle of 15° and their distribution was concentrated in the south. Satellite C08 was selected as the reference satellite due to it having the largest elevation angle.

First, based on the observations $\Delta P_{\left(0,1,1\right)}$ and $\Delta \phi_{\left(0,-1,1\right)}$, the GIF model was constructed to work out the EWL ambiguity $\Delta N_{\left(0,-1,1\right)}$ for a single epoch. The experiment showed that the ambiguity for each satellite was fixed accurately because the total error of each observation was much smaller than half a wavelength.

**Figure 6:**
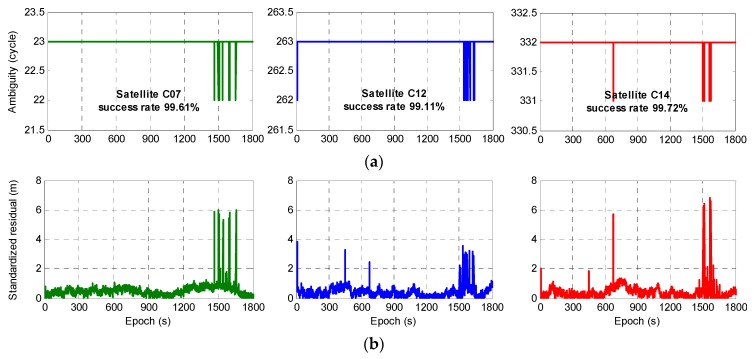
Performance of fixed ambiguity (**a**) and standardized residuals (**b**) for satellites C07, C12, and C14.

**Figure 7:**
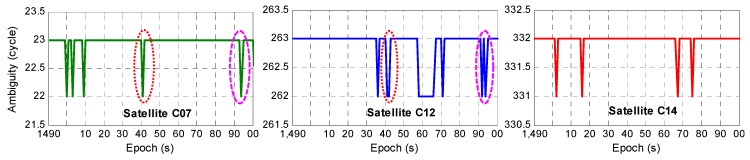
Fixed ambiguity from the 1490th to the 1600th epoch for satellites C07, C12, and C14.

As discussed in Section 2, several combined observations could be used to build a GF model for the EWL ambiguity $\Delta N_{\left(1,4,-5\right)}$ resolution. In this paper, the pseudo-range observation $\Delta P_{\left(1,0,0\right)}$ was taken as an example. The experiment showed that among 1800 epochs, there were 27 epochs in which the ambiguity was incorrect and the AR success rate was 98.5%. Due to the different levels of observation noise and ionospheric delays for each observation, the AR success rate for each satellite was also different. Except for the reference satellite, the ambiguity success rate for four satellites failed to reach 100%, among which only satellite C06 had an incorrect ambiguity in the 450th epoch, and the success rates for satellites C07, C12, and C14 were 99.61%, 99.11%, and 99.72%, respectively, as shown in Figure 6 (top). It can be seen that satellite C12 had an incorrect ambiguity in the first few epochs, and the other three satellites with incorrect ambiguities were concentrated mainly in the 1490th to 1600th epochs, as shown in detail in Figure 7; especially, satellites C07 and C12 had incorrect ambiguities at the same time in the 1541th and 1594th epochs, while in the remaining epochs, only one satellite had an incorrect ambiguity.

Based on the initial fixed ambiguity, given that the pseudo-range often exhibited a large amount of noise, the observations $\Delta \phi_{\left(0,-1,1\right)}$ and $\Delta \phi_{\left(1,4,-5\right)}$ were selected to estimate the DD ionospheric delay. Finally, the model for relative positioning for observation $\Delta \phi_{\left(1,4,-5\right)}$ was built with the estimated DD ionospheric delay and the initial fixed ambiguity. Figure 6b shows the performance of the standardized residuals for satellites C07, C12, and C14, and by considering of Figure 6a, it can be seen that when one of the satellites had the incorrect ambiguity in an epoch, that is, the observation contained a gross error, the corresponding standardized residual as abnormal. At the same time, the gross errors affected the standardized residuals for the other satellites to different degrees. For example, in the 679th epoch, the ambiguity for satellite C14 failed to be fixed. This gross error caused an error of more than 2 m in the standardized residual for satellite C12, and this result had no obvious influence on satellite C07. Moreover, the greatest influence was on its residual, which reached nearly 6 m. In the 11th epoch, the standardized residuals of satellite C12 changed greatly due to its incorrect ambiguity, and the performance was similar to that described above. Therefore, full use of this feature can be made for the validation of the reliability of ambiguity.

**Figure 8:**
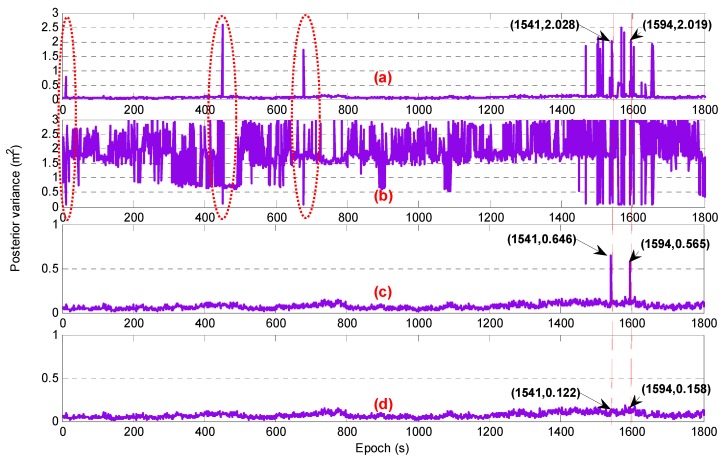
The performance of the posterior variance (**a**) with the initial ambiguity, (**b**) with candidate ambiguity during the first detection, (**c**) with the ambiguity after the first detection, and (**d**) with the final ambiguity.

The reliability of the initial ambiguity was validated epoch by epoch. First, assuming that the ambiguity with the maximum standardized residual was incorrect and the second-best ambiguity of this observation was selected while the other ambiguity values were unchanged, a new set of candidate ambiguities $\Delta {N^{\prime \prime }}_{\left(1,4,-5\right)}$ was reconstructed. After that, the new posterior variance was obtained through the LS method. Figure 8 shows the performance of the posterior variance during the gross error detection, where Figure 8a,b show the posterior variance for the initial ambiguity and candidate ambiguity, respectively. The optimal ambiguity could be chosen by comparing the two. From Figure 8a, it can be seen that as long as the ambiguity of one satellite was incorrect, the posterior variance was abnormal, and this value was larger than the value for the candidate ambiguity. Therefore, the candidate ambiguity $\Delta {N^{\prime \prime }}_{\left(1,4,-5\right)}$ was selected as the accurate value, thus the posterior variance was improved. Figure 8c shows the posterior variance after the first detection. Compared with Figure 8a, most of the outliers had disappeared. Particularly, as discussed above, in the 1541st and the 1594th epochs, there were two satellites whose ambiguities were incorrect; therefore, the posterior variance was still abnormal after the first gross detection, with the values being 0.646 m^2^ and 0.565 m^2^, respectively. According to a gross step-by-step detection process, based on the new standardized residual, the new maximum value may be found and its ambiguity may be checked using the same method. Finally, in this experiment, all incorrect ambiguities were corrected and the AR success rate reached 100%. Figure 8d shows the final value of the posterior variance.

### 6.2. Long Baseline with an Active Ionosphere

Considering that this method is affected to some extent by the ionospheric delay, the following experiment was carried out on 2 October 2019. Two observation stations, XM01 and LY01, were set up in Xiamen and Longyan city in Fujian province with a baseline distance of 116 km. The data collection started at noon local time and lasted for three hours with a 1 s sampling interval. There were a total of nine common-view and available BDS satellites with an elevation cut-off angle of 15°, and it was an active time for the ionosphere during the day

**Figure 9:**
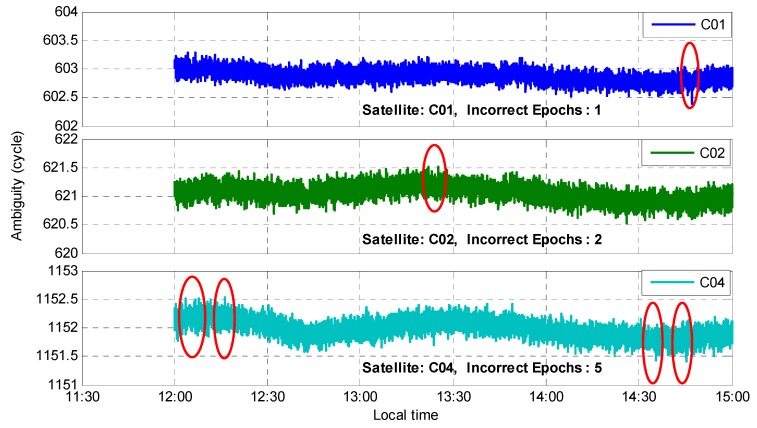
Performance of the float ambiguity for the satellites C01, C02, and C04.

The EWL ambiguity $\Delta N_{\left(1,4,-5\right)}$ was obtained by rounding with a single epoch. Except for the reference satellites, the remaining eight satellites had three satellites where the ambiguity success rate was not 100%. The three satellites were C01, C02, and C04, and the corresponding number of epochs with incorrect ambiguities were 1, 2, and 5, respectively. Figure 9 shows the performance of their float ambiguities. For all epochs, there were eight epochs where the ambiguity failed to be fixed such that the fixed ambiguity success rate was 99.92%. In particular, each epoch had only one satellite with an incorrect ambiguity.

**Figure 10:**
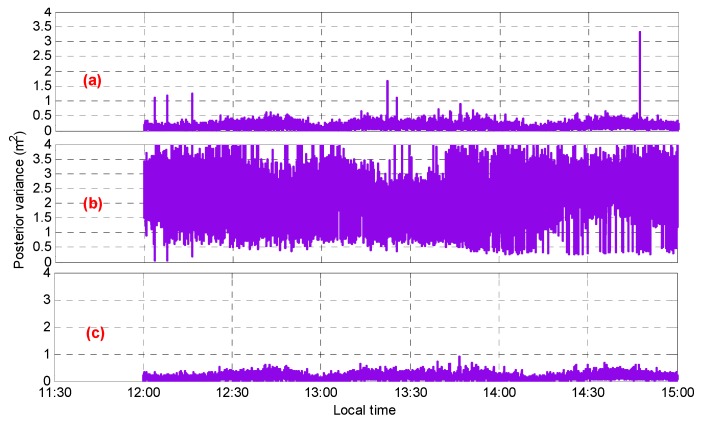
The performance for the posterior variance (**a**) with the initial ambiguity, (**b**) with the candidate ambiguity, and (**c**) with the final ambiguity.

**Figure 11:**
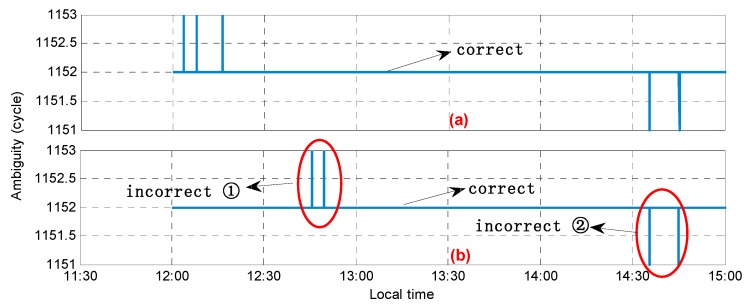
The performance for the fixed ambiguity for satellite C04 (**a**) without ambiguity validation and (**b**) with ambiguity validation.

Figure 10 shows the performance for the posterior variance during the gross error detection with Figure 10a,b showing the posterior variance with the initial and candidate ambiguities, respectively. In comparison with Figure 9, it can be seen that among the eight epochs mentioned above, six of them showed a large abnormal posterior variance; that is, the ambiguity had a wrong solution and the accurate values were obtained through gross error detection and correction. However, for satellite C04, in the two epochs with the incorrect ambiguity appearing after 14:30, the corresponding posterior variance did not appear abnormal and was less than the value corresponding to the candidate ambiguity (correct value); therefore, the gross error (ambiguity validation) detection failed. Figure 11 shows the performance for the fixed ambiguity of satellite C04 with and without ambiguity validation. It was found that in addition to the above missing detection, in Figure 11, there were also two epochs (incorrect ambiguity ①) with incorrect assessments; that is, the posterior variance corresponding to the candidate ambiguity (incorrect value) was less than the one with the initial ambiguity (correct value). In general, after the ambiguity validation, the ambiguity success rate for satellites C01 and C02 reached 100%, and for satellite C04, there were still four epochs with an incorrect ambiguity, and the ambiguity success rate increased to 99.96%.

### 6.3. Different Length Baselines Tests

To further analyze the applicability of the method, data for different periods in two different places were selected. One data set was from the Suzhou CORS in China, with three baselines of lengths 40 km, 52 km, and 66 km being selected, including the baseline discussed above. Similar to the above process, a total of 1800 epochs of data were collected with a 1 s sampling interval on 18 December 2014. The other data set was from the HeNan CORS in China, where three baselines of lengths 61 km, 104 km, and 175 km were selected. A total of 3600 epochs of data were collected on 1 March 2016, with a 1 s sampling interval and an elevation cut-off angle of 15°. Table 2 shows the AR success rate with and without reliability validation for the different baselines. It can be seen that without validation of the ambiguity reliability, only the success rate of the baseline ZKSQ-ZKLY (61 km) reached 100%, while the other five baselines were above 96%. The baseline XYXX-ZKSQ (104 km) had the lowest success rate, which was 96.82%; this meant that 115 out of 3600 epochs had an incorrect ambiguity. In general, after validation of the ambiguity reliability, the success rate was significantly improved. There were four baselines where the AR success rate reached 100% where the baseline length was 40 to 70 km; the success rate of the two longest baselines XYXX-ZKSQ (104 km) and XYXX-XCYL (175 km) did not reach 100%. However, compared with the initial ambiguity, the success rate had increased from 96.82% and 98.44% to 98.80% and 99.67%, respectively. It was found that during the gross error detection, the posterior variance with initial ambiguity (incorrect value) in these epochs was smaller than the one with the candidate ambiguity (correct value), which resulted in the incorrect ambiguity being selected. The main reason for the preliminary analysis was that the 4.519 m gross error caused by the incorrect ambiguity, which was just balanced out by some of the observed noise and the ionospheric delay, resulted in the residual sum of the squares being smaller than the one with the correct ambiguity.

## 7. Conclusions

Ambiguity reliability plays an important role in ensuring the accuracy and timeliness of GNSS high-precision positioning. The Chinese BDS is the first fully available triple-frequency GNSS system that provides positioning, navigation, and timing services independent of a GPS, and brings opportunities and challenges for GNSS positioning. Regarding the resolution and validation of EWL ambiguity $\Delta N_{\left(1,4,-5\right)}$, this research first compared and analyzed the AR performance for four different combined observations from both a theoretical basis and with measured data. The study showed that the GIF model constructed with optimal pseudo-range observation $\Delta P_{\left(-5,2,2.65\right)}$ had the lowest AR success rate, which was only 68.26%, while the ambiguities obtained by the other three observations gave approximately the same accuracy, with the success rates all being over 98%, but none reaching 100%. On this basis, because the AR for each satellite was relatively independent and the candidate ambiguity with high reliability usually had only two values, combined with the principle of gross error detection, a method for validating the reliability of the EWL ambiguity $\Delta N_{\left(1,4,-5\right)}$ using a single epoch was proposed.

The data for six different baselines were selected from HNCORS and SZCORS in China. This experiment showed that in a single epoch when only one or two satellites have incorrect ambiguities, the gross error caused by the incorrect ambiguity will affect the standardized residuals of all observations to different degrees whilst having the greatest influence on its observation. The ambiguity validation based on gross error detection can be used for a real-time EWL ambiguity check and correction using a single epoch in TCAR; especially for the medium baseline, the AR success rate after validation could reach 100%. For a long baseline, due to the increase in atmospheric error, the result was affected to a certain extent. However, compared with the initial ambiguity, the success rates increased from 96.82% and 98.44% to 98.80%, and 99.67%, respectively. Due to the limitations of gross error detection, the ambiguity validation method proposed in this paper applies to the condition where only one or two satellites have incorrect ambiguities in one epoch. Further research is needed on the case where multiple satellites have incorrect ambiguities for observations performed at the same time.

## Figures and Tables

**Table 1:** Characteristics of the extra-wide-lane (EWL) ambiguity $\Delta N_{\left(1,4,-5\right)}$ for different cases.

$\Delta P_{\left(i,j,k\right)}$ $\mathrm{or}\;\Delta \phi_{\left(i,j,k\right)}$	$\frac{\beta_{\left(1,4,-5\right)}\pm \beta_{\left(i,j,k\right)}}{\lambda_{\left(1,4,-5\right)}}\;(\mathrm{Cycle})$	$\sigma_{N_{\left(1,4,-5\right)}}\;(\mathrm{Cycle})$			
		Case 1 $\sigma_{\Delta \phi }=0.5\mathrm{cm}$ $\sigma_{\Delta P}=50\mathrm{cm}$	Case 2 $\sigma_{\Delta \phi }=0.5\mathrm{cm}$ $\sigma_{\Delta P}=100\mathrm{cm}$	Case 3 $\sigma_{\Delta \phi }=1.0\mathrm{cm}$ $\sigma_{\Delta P}=50\mathrm{cm}$	Case 4 $\sigma_{\Delta \phi }=1.0\mathrm{cm}$ $\sigma_{\Delta P}=100\mathrm{cm}$
$\Delta P_{\left(-5,2,2.65\right)}$	0	0.344	0.648	0.417	0.689
$\Delta P_{\left(1,0,0\right)}$	0.259	0.156	0.207	0.282	0.313
$\Delta P_{\left(0,1,1\right)}$	0.352	0.141	0.156	0.274	0.282
$\Delta \phi_{\left(0,-1,1\right)}$	0.352	0.137	0.137	0.275	0.275

**Table 2:** AR success rate with and without reliability validation for the different baselines.

Data Source	Reference Station	Baseline Length (km)	Without Checking	With Checking
SZCORS	TAOY-JINT	40	99.87%	100%
	JINT-WEIT	52	99.50%	100%
	WEIT-TAOY	66	98.50%	100%
HNCORS	ZKSQ-ZKLY	61	100%	100%
	XYXX-ZKSQ	104	96.82%	98.69%
	XYXX-XCYL	175	98.44%	99.69%
